# Резолюция совместного заседания экспертного совета «Гипопаратиреоз в эпоху сложных задач и прорывных технологий»

**DOI:** 10.14341/probl13805

**Published:** 2026-09-08

**Authors:** Н. Г. Мокрышева, Т. П. Бардымова, Е. Е. Бибик, А. К. Еремкина, Т. Л. Каронова, Е. В. Ковалева, И. В. Крюкова, И. П. Малая, В. А. Петеркова, Л. С. Созаева, М. Ш. Шамхалова, Т. В. Якимова

**Affiliations:** Национальный медицинский исследовательский центр эндокринологии им. академика И.И. Дедова Россия; Endocrinology Research Centre Russian Federation; Иркутская государственная медицинская академия последипломного образования Россия; Irkutsk State Medical Academy of Postgraduate Education Russian Federation; Национальный медицинский исследовательский центр им. В.А. Алмазова Россия; V.A. Almazov National Medical Research Center of Endocrinology Russian Federation; ГБУЗ МО МОНИКИ им. М.Ф. Владимирского или Московский областной научно-исследовательский клинический институт им. М.Ф. Владимирского Россия; Moscow Regional Research and Clinical Institute («MONIKI») Russian Federation; Республиканский клинический госпиталь для ветеранов войн Россия; Republican Clinical Hospital for War Veterans of the Chuvash Republic Ministry of Health Russian Federation

**Keywords:** гипопаратиреоз, орфанные заболевания, гормональная терапия, междисциплинарность, hypoparathyroidism, orphan diseases, hormonal therapy, interdisciplinarity

## Abstract

25 марта 2026 года в г. Москве в ФГБУ «НМИЦ эндокринологии им. академика И.И. Дедова» Минздрава России прошло заседание экспертного совета «Гипопаратиреоз в эпоху сложных задач и прорывных технологий», посвященное обсуждению вопросов эпидемиологии хронического гипопаратиреоза (ГПТ) в России, особенностям ведения пациентов с данной патологией в других странах, сходства и различия зарубежных и отечественных клинических рекомендаций.

Обсуждены успехи в совершенствовании диагностики и персонализированного лечения пациентов с ГПТ в России, а также необходимость внедрения передовых практик в практическое здравоохранение в рамках пересмотра клинических рекомендаций в 2027 г.

Обозначена важность включения хронического ГПТ в официальный перечень редких (орфанных) заболеваний, а также необходимость продолжения изучения особенностей заболевания и его осложнений, диагностики, мониторинга и профилактики ГПТ, в том числе в долгосрочной перспективе, и актуальность междисциплинарного профессионального альянса, позволяющего достигать оптимальных исходов лечения и улучшения качества жизни больных хроническим ГПТ.

Экспертами обозначен вопрос необходимости внедрения инновационных методов эффективного лечения, включая заместительную гормональную терапию ГПТ. В ходе Совета рассмотрены эффективность и безопасность новой терапевтической технологии «ТрансКон паратгормон». Приглашенный эксперт, доктор медицины профессор Университетской школы медицины Мармара, отделения эндокринологии и метаболизма Департамента внутренней медицины (Стамбул, Турция) Дилек Гогаш Явуз (Dilek Gogas Yavuz) поделилась опытом применения лекарственного препарата и результатами международных клинических исследований. Данная резолюция является кратким изложением вопросов, рассмотренных в выступлениях членов Совета и в процессе профессионального обсуждения.

## Abstract (en)

On March 25, 2026, a meeting of the Expert Council entitled "Hypoparathyroidism in the Era of Complex Challenges and Breakthrough Technologies" was held in Moscow at the Endocrinology Research Centre. The meeting focused on the epidemiology of chronic hypoparathyroidism (hypoPT) in Russia, the specifics of patient management with this pathology in other countries, and the similarities and differences between international and Russian clinical guidelines.

The meeting discussed advances in improving diagnostics and personalized treatment for patients with hypoPT in Russia, as well as the need to implement best practices in practical healthcare as part of the 2027 clinical guidelines revision.

The importance of including chronic hypoPT in the official list of rare (orphan) diseases was highlighted, as was the need to continue studying the characteristics of the disease and its complications, diagnosis, monitoring, and prevention of hypoPT, including in the long term. The meeting also highlighted the relevance of an interdisciplinary professional alliance to achieve optimal treatment outcomes and improve the quality of life of patients with chronic hypoPT. The experts highlighted the need to implement innovative, effective treatment methods, including hormone replacement therapy for hypoPT. The Council discussed the efficacy and safety of the new therapeutic technology, TransCon parathyroid hormone. Invited expert Dilek Gogas Yavuz, MD, Professor at the Marmara University School of Medicine, Department of Endocrinology and Metabolism, Department of Internal Medicine (Istanbul, Turkey), shared her experience with the drug and the results of international clinical trials. This resolution is a summary of the issues discussed in the Council members’ presentations and during the professional discussions.

## ГИПОПАРАТИРЕОЗ В РОССИИ: СОВРЕМЕННОЕ СОСТОЯНИЕ ПРОБЛЕМЫ И НЕРЕШЕННЫЕ ВОПРОСЫ

В 2018 г. в ГНЦ РФ ФГБУ «НМИЦ эндокринологии им. академика И.И. Дедова» Минздрава России под непосредственным руководством Н.Г. Мокрышевой началась работа по созданию реестра пациентов с гипопаратиреозом (ГПТ), включая случаи редких наследственных синдромов. В 2020 г. проект трансформирован в формат всероссийской Базы данных клинико-эпидемиологического мониторинга пациентов с хроническим ГПТ с возможностью дистанционного доступа из различных регионов РФ. В настоящее время База данных включает динамически обновляемые сведения о более чем 1900 пациентах из 81 региона России, что сопоставимо и даже превышает отдельные ведущие зарубежные базы данных по указанному заболеванию. В Базу данных вносится информация о пациентах, обратившихся по поводу ГПТ за амбулаторной помощью или госпитализированных в стационар лечебного учреждения субъекта РФ. Среди наиболее активных субъектов РФ по ведению Базы данных стоит отметить следующие регионы: Воронежскую, Иркутскую, Саратовскую и Нижегородскую области, Чувашскую Республику и Забайкальский край [1][2].

Активная работа под эгидой ГНЦ РФ ФГБУ «НМИЦ эндокринологии им. академика И.И. Дедова» Минздрава России позволила оценить клинико-демографические характеристики патологии, эффективность различных комбинаций лекарственных препаратов, а также отследить течение заболевания и исходы у пациентов с различными формами ГПТ [1–4].

Последний комплексный анализ Базы данных с включением 1776 пациентов из 81 субъекта РФ проведен в 2024 г. В исследуемой популяции хронический ГПТ преобладал среди женщин (86,5%), большинство пациентов имели послеоперационную этиологию заболевания (70,1%), при этом наиболее часто хронический ГПТ развивался вследствие хирургических вмешательств на органах шеи по поводу высокодифференцированного рака щитовидной железы (ВДРЩЖ) (44,1%). Нехирургический ГПТ суммарно был диагностирован у 531 пациента (30,0%), из которых идиопатический — у 390 (73,4%), в рамках аутоиммунного полигландулярного синдрома 1 типа (АПС-1) — у 80 (15,1%), другие синдромальные формы ГПТ (ассоциированные с генетическими мутациями, за исключением гена AIRE) наблюдались у 15 человек (2,8%).

Целевые значения кальциемии были достигнуты у 44,6% пациентов, фосфатемии — у 54,7%. При анализе ассоциированных осложнений структурная патология почек в виде нефрокальциноза и нефролитиаза наблюдалась у 33,4 и 10,7% соответственно; снижение расчетной скорости клубочковой фильтрации (рСКФ) до хронической болезни почек 3а-5 стадий — у 17,4%. Катаракта диагностирована в 34,7% случаев. Анализ получаемой терапии показал, что более 70,4% пациентов получали «классическую» комбинацию препаратов активных метаболитов витамина D и кальция. Дополнительные препараты (магния, рекомбинантного человеческого паратгормона (рчПТГ), тиазидные диуретики) назначались в 5,9% случаев [1][2].

В 2021 г. была разработана и в последующем внедрена система поддержки принятия врачебных решений (СППВР) — информационная система, предназначенная для помощи практикующим специалистам в решении клинических задач. СППВР базируется на утвержденных федеральных клинических рекомендациях и позволяет на основании анализа внесенной информации о пациенте сообщить пользователям о ее корректности, наличии/отсутствии осложнений, оценить назначенную схему терапии, предупредив о недопустимых комбинациях препаратов, а также напомнить о необходимости дообследования пациента [5].

В настоящее время на базе специализированного отделения разработана уникальная математическая модель «Способ индивидуализированного подбора и прогнозирования доз лекарственных препаратов для лечения пациентов с хроническим ГПТ». Данная модель представляет собой СППВР на основании ансамблевого метода машинного обучения (ExtraTreesRegressor). Разработанный алгоритм позволяет прогнозировать изменения дозы препаратов альфакальцидола и/или карбоната кальция и/или колекальциферола на основе анализа обработанных данных и дает рекомендации по необходимой корректировке терапии для достижения целевого уровня общего кальция в крови [6]. После валидации модель будет также интегрирована в клиническую практику.

Расширение Базы данных крайне важно для пополнения сведений о распространенности и заболеваемости ГПТ в РФ. Изучение анамнестических, лабораторно-инструментальных характеристик заболевания у пациентов в российской популяции — важный этап на пути оптимизации лечения и диагностики. Проведенный анализ указывает на недостаточный лабораторный контроль за заболеванием, а также охват пациентов в отношении скрининга долгосрочных осложнений. Решение указанной проблемы возможно путем совершенствования действующих клинических рекомендаций, а также повышения осведомленности врачей и пациентов.

В феврале 2026 г. согласно решению Минздрава России тяжелые формы хронического ГПТ (коды по МКБ-10: Е20.0, Е20.8, Е20.9, Е31.0, Е89.2) признаны в России редким (орфанным) заболеванием, что позволяет этой группе пациентов рассчитывать на поддержку государства в получении необходимого дорогостоящего лечения [7].

Проведенный в 2024–2025 гг. в шести регионах России пилотный скрининг кальциемии показал высокую распространенность гипокальциемии (до 20%) среди населения. Для своевременной диагностики патологии минерального обмена, в том числе ГПТ, целесообразно внедрить массовое скрининговое исследование кальция крови в рамках диспансеризации населения России.

По мнению экспертов, в настоящее время основными направлениями дальнейшей работы в данной области являются:

- необходимость внедрения скрининга кальциемии и повышение доступности анализа кальция крови в рамках диспансеризации населения России;

- пополнение эпидемиологических данных по ГПТ, подключение новых медицинских учреждений к ведению Базы данных;

- повышение информированности врачей и пациентов о заболевании, в том числе о рисках его развития после операций в области шеи, проведение образовательных мероприятий и научно-практических конференций, как во врачебном, так и пациентском сообществах;

- активный мониторинг пациентов с ежегодным проведением необходимых лабораторных и инструментальных исследований согласно действующим клиническим рекомендациям.

## НОВОЕ В МЕЖДУНАРОДНЫХ КЛИНИЧЕСКИХ РЕКОМЕНДАЦИЯХ ПО ЛЕЧЕНИЮ ГИПОПАРАТИРЕОЗА

Первые клинические рекомендации по ГПТ были приняты на первой международной конференции по ГПТ, проведенной во Флоренции в мае 2015 г. Европейским сообществом эндокринологов (European society of endocrinology, ESE) и Американской ассоциацией клинических эндокринологов (American association of clinical endocrinologists, AACE) [8]. Обновления произошли в 2025 г., так как за этот период времени сильно изменились методы ведения пациентов, а также были накоплены знания о тяжести этого заболевания и его осложнений, появились прорывные медицинские технологии [9].

В соответствии с положениями клинических рекомендаций ESE 2025 г. основные принципы ведения пациента с ГПТ заключаются в персонализированном подходе с оптимизацией качества жизни, минимизацией симптомов гипокальциемии и улучшением долгосрочного прогноза. Лечение должно быть индивидуальным и ориентированным на общее самочувствие пациента, а решения должны приниматься с учетом индивидуальной реакции пациента и клинических симптомов. Принципиальным отличием от руководства 2015 г. стало более активное обсуждение рекомбинантных форм ПТГ в лечении пациентов с хроническим ГПТ. Руководство 2025 г. рекомендует заместительную терапию ПТГ у лиц с хроническим ГПТ, у которых сохраняются признаки или симптомы заболевания, несмотря на оптимизацию лечения с помощью традиционной терапии, включая стойкую гиперфосфатемию и гиперкальциурию, а также отмечается ухудшение качества жизни и снижение функции почек [9].

Стоит отметить, что возможности рекомбинантной терапии ПТГ также обсуждаются другими эндокринологическими сообществами. Группа восточноевропейских экспертов (Misiorowski et al.) рекомендует рассматривать возможность заместительной терапии препаратами ПТГ у пациентов, у которых на стандартном лечении заболевание не поддается адекватному контролю [10]. В итальянском руководстве (Guidelines on the therapeutic management of post-surgical hypoparathyroidism in adults, Istituto Superiore di Sanità, 2025) указано, что терапия рчПТГ позволяет достичь стойкой нормокальциемии, стабилизировать уровень фосфора крови, улучшить общее состояние пациентов и позволяет им в большинстве случаев уменьшить зависимость или стать независимыми от стандартной терапии [11]. Согласно рекомендациям турецкого профессионального сообщества (Turkish Society of Endocrinology and Metabolism Guidelines for the Diagnosis and Treatment of Osteoporosis and Metabolic Bone Diseases, 2025), заместительная терапия препаратами ПТГ может быть назначена пациентам, которые не могут достичь целевых показателей при стандартном лечении, плохо переносят его или у которых развиваются острые или хронические осложнения основного заболевания [12].

Таким образом, большинство международных экспертных сообществ сходятся во мнении, что лечение хронического ГПТ должно быть персонализированным и ориентированным на качество жизни и долгосрочный прогноз пациентов. Заместительная терапия рчПТГ способствует увеличению эндогенного активированного витамина D, восстанавливает почечную реабсорбцию кальция и выведение фосфатов с мочой, что позволяет достичь оптимального компромисса между минимизацией симптомов гипокальциемии и риском побочных эффектов лечения, таких как гиперкальциурия или гиперфосфатемия.

## ПОДХОДЫ К ДИАГНОСТИКЕ И МОНИТОРИНГУ ГИПОПАРАТИРЕОЗА И ЕГО ОСЛОЖНЕНИЙ В РОССИИ. АКТУАЛЬНОСТЬ ИННОВАЦИЙ И МЕЖДИСЦИПЛИНАРНОГО АЛЬЯНСА

В настоящее время преобладающей формой заболевания остается хронический послеоперационный ГПТ (более 70% пациентов), диагноз устанавливают на основании сохранения гипокальциемии в сочетании с неадекватно низким показателем паратгормона при отсутствии лекарственной терапии спустя 6 и более месяцев после хирургического вмешательства на органах шеи [13]. Однако у части больных (до 7,4%) после операции функциональная активность околощитовидных желез восстанавливается в течение 12-ти и даже более месяцев после операции, в связи с чем вопрос о сроке установления диагноза хронического ГПТ остается дискутабельным и требует пересмотра в новом проекте клинических рекомендаций в 2027 г.

Особое внимание уделяется проблеме «неконтролируемого» ГПТ, связанной не только с трудностями достижения целевых показателей кальций-фосфорного обмена, но и с долгосрочными осложнениями заболевания и его терапии, сохраняющимися симптомами гипокальциемии и снижением качества жизни пациентов.

Для стратификации пациентов важно учитывать общепринятые целевые диапазоны параметров (концентрации кальция, фосфора крови, суточной кальциурии) для оценки компенсации ГПТ. Диагностика состояний компенсации и декомпенсации обычно не вызывает затруднений, тогда как группа субкомпенсации остается наименее изученной и представляет сложность для клинической оценки. В связи с этим научный и практический интерес представляет изучение группы пациентов в стадии субкомпенсации, где нормокальциемия сочетается с гиперфосфатемией и/или превышением целевых показателей кальция в суточной моче. Выделение специфических лабораторных изменений у этой категории пациентов позволит оптимизировать динамическое наблюдение и своевременно корректировать терапию для профилактики поздних осложнений.

Важно отметить, что особое внимание к группе с субкомпенсацией обусловлено также необходимостью выявления «скрытых» эпизодов гипо-/гиперкальциемий и персонализированного подбора терапии с указанием индивидуальной кратности и времени приема препаратов. Достижение этой цели возможно благодаря разработке и внедрению в клиническую практику суточного профиля кальциемии (12-кратное исследование уровня альбумин-скорректированного кальция крови в сутки) [14]. Данный подход является инновационным и был интегрирован в клинические рекомендации по ГПТ при пересмотре в 2025 г. [13].

Помимо регулярного лабораторного контроля, пациентам с хроническим ГПТ рекомендуется периодическая оценка структурных и функциональных изменений почек, а также оценка органов зрения. Неоптимальный лабораторный контроль, эпизоды гипо-и гиперкальциемии, гиперкальциурии, гиперфосфатемии способствуют развитию и прогрессированию и других ассоциированных осложнений (кальцификация структур головного мозга и неврологические нарушения, патология сердечно-сосудистой системы и др.). Это диктует необходимость формирования междисциплинарной врачебной команды для оптимизации ведения пациентов с ГПТ.

Большой проблемой остается неудовлетворительное качество жизни большей части пациентов с хроническим ГПТ, их низкая социальная активность. По результатам российского исследования, пациенты с ГПТ имеют сниженные показатели качества жизни по опроснику SF-36 и большую склонность к развитию астении по опроснику MFI-20. В основном на качество жизни пациентов влияет количество принимаемых таблеток в сутки, что согласуется с результатами зарубежных исследований [15–19]. Также отмечено, что длительность заболевания сама по себе не способствовала снижению качества жизни, а напротив, была ассоциирована с хорошими показателями, что может быть объяснено большей осведомленностью о течении заболевания и формированием адаптации пациентов к нему. В связи с этим создание и проведение образовательных школ для пациентов является актуальным направлением работы медицинских специалистов.

Эксперты сошлись во мнении, что для оптимизации диагностики и долгосрочного мониторинга пациентов с ГПТ целесообразно:

- пересмотреть определение хронического послеоперационного ГПТ с установлением диагноза при сохранении гипокальциемии в сочетании с неадекватно низким показателем ПТГ спустя 12 и более месяцев после хирургического вмешательства на органах шеи;

- адаптировать лечение пациентов с ГПТ на поддержание целевых показателей кальций-фосфорного обмена во избежание развития поздних осложнений (уровня альбумин-скорректированного кальция на нижней границе референсного диапазона лаборатории у пациентов без клинических симптомов гипокальциемии, сывороточного фосфора в пределах референсного диапазона лаборатории, суточной экскреции кальция с учетом гендерных различий, 25(ОН) витамина D как в общей популяции);

- проводить суточное мониторирование кальция крови в случаях диагностики субкомпенсации заболевания для подбора персонализированной терапии;

- продолжить изучение спектра сердечно-сосудистой, психо-неврологической и костной патологии у пациентов с ГПТ для уточнения факторов, способствующих их развитию и прогрессированию;

- сформировать междисциплинарную команду специалистов для согласования лечения пациентов с длительным осложненным течением ГПТ;

- проводить образовательные школы для врачей и пациентов с хроническим ГПТ, направленные на поддержание достаточного уровня их информированности о заболевании и приемлемого качества жизни.

## ПОДХОДЫ К ЛЕЧЕНИЮ ГИПОПАРАТИРЕОЗА В РОССИИ

Классическая схема терапии хронического ГПТ представляет собой комбинацию пероральных препаратов витамина D и его аналогов и солей кальция. Цель терапии — достижение оптимальных значений кальциемии и фосфатемии в условиях недостаточного поступления кальция с пищей. Для коррекции гиперкальциурии в некоторых случаях могут применяться тиазидные диуретики. Средние суточные дозы альфакальцидола/кальцитриола у большинства (до 93%) пациентов составляют 0,5–3,0 мкг/сут, препаратов кальция — 1000–2000 мг/сут [13].

Терапия препаратами витамина D и его производными в сочетании с препаратами кальция способствует уменьшению симптомов гипокальциемии. Однако порядка 10–15% пациентов не достигают компенсации состояния даже на фоне приема рекомендованных доз препаратов витамина D и кальция (препараты кальция ≥3000 мг/сут, препараты альфакальцидола ≥4 мкг/сут, кальцитриола ≥2 мкг/сут). Дальнейшее увеличение доз препаратов ассоциировано как со снижением качества жизни пациентов (прием большого качества препаратов в течение дня), так и с повышением риска долгосрочных осложнений ГПТ. Предрасполагающими факторами к неудовлетворительной компенсации ГПТ являются синдром мальабсорбции, высокие дозы препаратов кальция, способствующие констипации. При некоторых генетически обусловленных и редких формах заболевания (аутоиммунном ГПТ, синдроме Ди-Джорджи, синдроме Бараката, синдроме Саньяд-Сакати и др.) достижение устойчивой компенсации остается сложной клинической задачей. Для этих пациентов характерна тяжелая гипокальциемия с жизнеугрожающими гипокальциемическими кризами, они нуждаются в постоянном наблюдении специалистов, частых госпитализациях, теряют трудоспособность.

По экспертным расчетам, исходя из данных Росстата и Базы данных клинико-эпидемиологического мониторинга, ожидаемое количество пациентов с тяжелыми формами хронического ГПТ (включая аутоиммунный изолированный ГПТ, генетические варианты изолированного ГПТ, ГПТ в составе поликомпонентных генетических заболеваний, идиопатический ГПТ и хронический послеоперационный ГПТ, не контролируемый на стандартной терапии) составляет 2,99–4,8 случая на 100 000 населения.

В случае тяжелых форм заболевания в настоящее время по решению врачебной комиссии специализированного центра может быть рассмотрен вопрос о назначении терипаратида. Терипаратид — единственный доступный на сегодняшний день в России препарат рчПТГ, зарегистрированный в качестве средства анаболической антиостеопоротической терапии. Накоплены данные долгосрочного применения терипаратида у взрослых и детей с хроническим ГПТ. Терапия рчПТГ способствовала снижению потребности в препаратах витамина D и кальция, положительной динамике маркеров костного ремоделирования [20–23]. Однако оценка безопасности препарата в разных возрастных группах нуждается в уточнении посредством проведения крупных рандомизированных клинических исследований.

Перспективной и патогенетически обоснованной терапией для таких пациентов могут стать дорогостоящие препараты рчПТГ/агонисты рецептора ПТГ. Однако эти препараты в РФ не зарегистрированы, что существенно ограничивает их доступность для пациентов. Внесение тяжелых форм хронического послеоперационного и генетически обусловленного ГПТ в официальный перечень орфанных заболеваний в 2026 г. позволит обеспечить пациентам своевременное и адекватное лечение, а также повысит качество и продолжительность их жизни.

В рамках совершенствования лечения пациентов с ГПТ эксперты предлагают:

- обеспечить государственную поддержку пациентам с тяжелыми формами хронического ГПТ, в том числе в беспрепятственном обеспечении их дорогостоящими лекарственными препаратами рчПТГ;

- закрепить право назначения и инициации терапии препаратами рчПТГ специализированным отделениям ведущих научно-исследовательских эндокринологических центров РФ;

- расширить участие в международных клинических исследованиях новых лекарственных препаратов для заместительной терапии ГПТ, в том числе среди детской популяции пациентов.

## НОВАЯ ТЕХНОЛОГИЯ И НОВЫЕ ВОЗМОЖНОСТИ ТЕРАПИИ ГИПОПАРАТИРЕОЗА: ЗАРУБЕЖНЫЙ ОПЫТ. ТРАНСКОН ПТГ: ОБЗОР РЕЗУЛЬТАТОВ КЛИНИЧЕСКИХ ИССЛЕДОВАНИЙ

В рамках Совета было представлено сообщение приглашенного международного эксперта профессора Дилек Гогаш Явуз (Турция), в котором она поделилась международным опытом заместительной терапии, результатами клинических исследований и накопленным личным опытом успешного ведения пациентов с хроническим ГПТ с применением инновационного аналога ПТГ (препарат палопегтерипаратид, ТрансКон ПТГ).

Благодаря инновационной технологии ТрансКон (ориг. TransCon — transient conjugation) биологические свойства (строение, кинетика) препарата обеспечивают его эффективное действие при однократном ежедневном применении. ТрансКон-технология — уникальный механизм временного связывания инертного носителя с исходным лекарственным средством известного биологического действия. Палопегтерипаратид — это молекула-предшественник с замедленным высвобождением активного ПТГ1-34 предназначенная для поддержания концентрации ПТГ в крови в физиологическом диапазоне в течение 24 часов. После подкожного введения палопегтерипаратида свободный ПТГ медленно высвобождается в системный кровоток, обеспечивая его стабильную концентрацию в крови в течение суток, что приводит к поддержанию адекватной концентрации кальция крови и устраняет необходимость применения активных форм витамина D и препаратов кальция [24].

В рандомизированном двойном слепом плацебо-контролируемом исследовании 2-й фазы PaTH Forward оценивалась эффективность палопегтерипаратида (TransCon PTH) у 59 пациентов с ГПТ (послеоперационным, аутоиммунным, генетическим или идиопатическим). Все участники исходно получали активный метаболит витамина D и препараты кальция. Дизайн исследования предполагал 4-недельный слепой период (дозы препарата 15, 18, 21 мкг или плацебо) и далее 22-недельное открытое продление с коррекцией терапии. Первичной конечной точкой в конце 4-недельного слепого периода являлось стойкое поддержание нормокальциемии, уменьшение степени кальциурии в утренней порции мочи, а также снижение не менее чем на 50% или прекращение приема всех активных добавок витамина D и сокращение приема кальция максимум до 1000 мг/день. К 4-й неделе 50% пациентов на TransCon PTH достигли цели (нормокальциемия, снижение кальциурии, отмена/сокращение витамина D и кальция ≤1000 мг/сут) против 15% на плацебо (p<0,03), а 50% полностью отказались от активного витамина D и снизили потребление кальция. К 26-й неделе 91% участников полностью прекратили прием активного витамина D и снизили потребление кальция (≤500 мг/сут). 76% полностью отказались от всех препаратов. Также отмечалось снижение кальциурии с 415 мг/сут до 178 мг/сут (n=44), при сохранении нормофосфатемии, улучшение качества жизни согласно опроснику SF-36. Серьезных побочных эффектов или случаев прекращения лечения из-за нежелательных явлений не отмечалось [25].

По результатам исследования третьей фазы PaTHway Trial 93% (57/61) участников, получавших лечение изучаемым препаратом, смогли отказаться от стандартной терапии ГПТ — активных аналогов витамина D и препаратов кальция в терапевтических дозах. Использование палопегтерипаратида было ассоциировано с достижением физиологичных значений суточной кальциурии, а также с лучшими результатами показателей, отражающих качество жизни [26][27].

Таким образом, палопегтерипаратид в настоящее время является единственным доступным аналогом ПТГ, одобренным EMA и FDA для лечения хронического ГПТ. Отмечаются значительные положительные эффекты лечения данным препаратом, в первую очередь коррекция гиперкальциурии и гиперфосфатемии. Тем не менее, по результатам клинических испытаний и рутинной врачебной практики, международная рабочая группа по разработке клинических рекомендаций признает, что долгосрочные данные о безопасности и исходах (например, о частоте переломов, состоянии сердечно-сосудистой системы, почек) пока отсутствуют.

В рамках новой технологии терапии ГПТ ТрансКон ПТГ эксперты предлагают:

- при дальнейших клинических исследованиях акцентировать внимание на безопасности и долгосрочных последствиях применения препарата, в первую очередь со стороны сердечно-сосудистой системы и костной ткани, в том числе риске низкоэнергетических переломов;

- отслеживать опыт долгосрочного применения препарата в реальной клинической практике;

- расширить участие в международных клинических исследованиях, в том числе на детской популяции пациентов с ГПТ.

## ПЕРСПЕКТИВЫ ПРИМЕНЕНИЯ ИННОВАЦИОННОЙ ГОРМОНОЗАМЕСТИТЕЛЬНОЙ ТЕРАПИИ ГПТ В РОССИИ

Экспертами единогласно отмечено, что заместительная терапия ГПТ имеет несомненное преимущество перед стандартной терапией препаратами витамина D и его аналогами и препаратами кальция (симптоматическое лечение), как в отношении поддержания целевых показателей кальций-фосфорного обмена, так и влияния на субъективное самочувствие и качество жизни пациентов. По аналогии с инсулинозависимым сахарным диабетом хронический ГПТ предполагает пожизненное лечение, в связи с чем пациенты, особенно с тяжелыми формами заболевания, нуждаются в государственной поддержке по обеспечению лекарственными препаратами.

В связи с отсутствием отечественных препаратов ПТГ важным является расширение международного сотрудничества по проблеме хронического ГПТ, накопление опыта проведения международных клинических исследований применения лекарственных препаратов как среди взрослых, так и среди детей. ТрансКон ПТГ палопегтерипаратид, одобренный EMA и FDA, в настоящее время может являться наиболее перспективным лекарственным средством для заместительной терапии хронического ГПТ в России.

## ЗАКЛЮЧЕНИЕ И РЕЗЮМЕ ПО ИТОГАМ СОВМЕСТНОГО ЗАСЕДАНИЯ

По обсужденным вопросам хронического ГПТ эксперты пришли к следующим выводам и заключениям.

## ДОПОЛНИТЕЛЬНАЯ ИНФОРМАЦИЯ

Источники финансирования. Работа выполнена по инициативе авторов без привлечения финансирования.

Конфликт интересов. Авторы декларируют отсутствие явных и потенциальных конфликтов интересов, связанных с содержанием настоящей статьи.

Участие авторов. Все авторы одобрили финальную версию статьи перед публикацией, выразили согласие нести ответственность за все аспекты работы, подразумевающую надлежащее изучение и решение вопросов, связанных с точностью или добросовестностью любой части работы.

## References

[cit1] Kovaleva E. V., Salimkhanov R. K., Elfimova A. R., Eremkina A. K., Pershina-Miliutina A. P., Bibik E. E., Gorbacheva A. M., Vikulova O. K., Mokrysheva N. G. (2024). Complications of chronic hypoparathyroidism according to analysis database Russian Registry. Clinical and experimental thyroidology.

[cit2] Salimkhanov R. Kh., Kovaleva E. V., Elfimova A. R., Eremkina A. K., Pershina-Miliutina A. P., Bibik E. E., Gorbacheva A. M., Vikulova O. K., Mokrysheva N. G. (2024). Clinical profile of patients with chronic hypoparathyroidism according to the All-Russian registry. Clinical and experimental thyroidology.

[cit3] Kovaleva Elena V., Eremkina Anna K., Elfimova Alina R., Krupinova Julia A., Bibik Ekaterina E., Maganeva Irina S., Gorbacheva Anna M., Dobreva Ekaterina A., Melnichenko Galina A., Mokrysheva Natalia G. (2022). The Russian Registry of Chronic Hypoparathyroidism. Frontiers in Endocrinology.

[cit4] Khronicheskii gipoparatireoz: prediktory razvitiya oslozhnenii zabolevaniya i personalizatsiya vedeniya patsientov. Kovaleva E.V. — Diss. na soiskanie uch. st. k.m.n., 2022 g.

[cit5] Kovaleva E. V., Eremkina A. K., Ajnetdinova A. R., Miliutina A. P., Mokrysheva N. G. (2021). The Russian registry of chronic hypoparathyroidism and clinical decision support system integration. Problems of Endocrinology.

[cit6] Sposob individualizirovannogo podbora doz lekarstvennykh preparatov dlya lecheniya patsientov s khronicheskim gipoparatireozom. Mokrysheva Natal'ya Georgievna, Eremkina Anna Konstantinovna, Kovaleva Elena Vladimirovna, Elfimova Alina Rinatovna, Pershina-Milyutina Anastasiya Pavlovna. Patent na izobretenie №2861630, 6 maya 2026 g.

[cit7] Perechen' redkikh (orfannykh) zabolevanii, Minzdrav Rossii, ot 14 aprelya 2026 goda, https://minzdrav.gov.ru/documents/9863-perechen-redkih-orfannyh-zabolevaniy.

[cit8] Bollerslev Jens, Rejnmark Lars, Marcocci Claudio, Shoback Dolores M, Sitges-Serra Antonio, van Biesen Wim, Dekkers Olaf M (2015). European Society of Endocrinology Clinical Guideline: Treatment of chronic hypoparathyroidism in adults. European Journal of Endocrinology.

[cit9] Bollerslev Jens, Buch Ottilia, Cardoso Luís Miguel, Gittoes Neil, Houillier Pascal, van Hulsteijn Leonie, Makay Ozer, Marcocci Claudio, Pallais J Carl, Pilz Stefan, Rejnmark Lars, Yavropoulou Maria, Dekkers Olaf M (2025). Revised European Society of Endocrinology Clinical Practice Guideline: Treatment of Chronic Hypoparathyroidism in Adults. European Journal of Endocrinology.

[cit10] Misiorowski Waldemar, Kos-Kudła Beata, Hubalewska-Dydejczyk Alicja, Ruchała Marek, Zgliczyński Wojciech, Zygmunt Arkadiusz, Bilezikian John P. (2026). Treatment of hypoparathyroidism (HypoPT): Position Statement of the Expert Group for Polish Society of Endocrinology — update 2026. Endokrynologia Polska.

[cit11] Guidelines on the therapeutic management of post-surgical hypoparathyroidism in adults. Association of Endocrinologists (AME), Italian Society of Endocrinology (SIE), Italian Society of Orthopedics and Traumatology (SIOT), Italian Society of Osteoporosis, Mineral Metabolism and Skeletal Diseases (SIOMMMS), Italian Society of Endocrine Surgery (SIUEC), Istituto Superiore di Sanità, 2025

[cit12] Guidelines for the Diagnosis and Treatment of Osteoporosis and Metabolic Bone Diseases. Turkish Society of Endocrinology and Metabolism, 2025

[cit13] Klinicheskie rekomendatsii po gipoparatireozu u vzroslykh. Ministerstvo zdravookhraneniya Rossiiskoi Federatsii, 2025 g.

[cit14] Kovaleva E. V., Eremkina A. K., Mokrysheva N. G. (2021). Daily calcium profile in diagnosis of hypo- and hypercalcemia in patients with chronic hypoparathyroidism. clinical case series. Obesity and metabolism.

[cit15] Kovaleva E. V., Eremkina A. K., Mokrysheva N. G. (2023). Chronic hypoparathyroidism: clinical manifestations, complications and impact on the quality of life. FOCUS. Endocrinology.

[cit16] Arlt W, Fremerey C, Callies F, Reincke M, Schneider P, Timmermann W, Allolio B (2005). Well-being, mood and calcium homeostasis in patients with hypoparathyroidism receiving standard treatment with calcium and vitamin D. European Journal of Endocrinology.

[cit17] Sikjaer Tanja, Moser Emil, Rolighed Lars, Underbjerg Line, Bislev Lise Sofie, Mosekilde Leif, Rejnmark Lars (2016). Concurrent Hypoparathyroidism Is Associated With Impaired Physical Function and Quality of Life in Hypothyroidism. Journal of Bone and Mineral Research.

[cit18] Underbjerg L., Sikjaer T., Rejnmark L. (2018). Health‐related quality of life in patients with nonsurgical hypoparathyroidism and pseudohypoparathyroidism. Clinical Endocrinology.

[cit19] Siggelkow Heide, Clarke Bart L., Germak John, Marelli Claudio, Chen Kristina, Dahl‐Hansen Helen, Glenister Elizabeth, Bent‐Ennakhil Nawal, Judge Davneet, Mycock Katie, Bollerslev Jens (2019). Burden of illness in not adequately controlled chronic hypoparathyroidism: Findings from a 13‐country patient and caregiver survey. Clinical Endocrinology.

[cit20] Winer Karen K., Ko Chia Wen, Reynolds James C., Dowdy Karen, Keil Meg, Peterson Donna, Gerber Lynn H., McGarvey Charles, Cutler Gordon B. (2003). Long-Term Treatment of Hypoparathyroidism: A Randomized Controlled Study Comparing Parathyroid Hormone-(1–34) Versus Calcitriol and Calcium. The Journal of Clinical Endocrinology & Metabolism.

[cit21] Marcucci Gemma, Masi Laura, Cianferotti Luisella, Giusti Francesca, Fossi Caterina, Parri Simone, Gronchi Giorgio, Brandi Maria Luisa (2021). Chronic hypoparathyroidism and treatment with teriparatide. Endocrine.

[cit22] Winer Karen K., Sinaii Ninet, Peterson Donna, Sainz Bruno, Cutler Gordon B. (2008). Effects of Once Versus Twice-Daily Parathyroid Hormone 1–34 Therapy in Children with Hypoparathyroidism. The Journal of Clinical Endocrinology & Metabolism.

[cit23] Winer Karen K., Kelly Andrea, Johns Alicia, Zhang Bo, Dowdy Karen, Kim Lauren, Reynolds James C., Albert Paul S., Cutler Gordon B. (2018). Long-Term Parathyroid Hormone 1-34 Replacement Therapy in Children with Hypoparathyroidism. The Journal of Pediatrics.

[cit24] Karpf DB, Pihl S, Mourya S, Mortensen E, Kovoor E, Markova D, Leff JA. A Randomized Double-Blind Placebo-Controlled First-In-Human Phase 1 Trial of TransCon PTH in Healthy Adults. J Bone Miner Res. 2020;35(8):1430-1440. doi: https://doi.org/10.1002/jbmr.4016 PMC9328939 32212275

[cit25] Khan Aliya A, Rejnmark Lars, Rubin Mishaela, Schwarz Peter, Vokes Tamara, Clarke Bart, Ahmed Intekhab, Hofbauer Lorenz, Marcocci Claudio, Pagotto Uberto, Palermo Andrea, Eriksen Erik, Brod Meryl, Markova Denka, Smith Alden, Pihl Susanne, Mourya Sanchita, Karpf David B, Shu Aimee D (2021). PaTH Forward: A Randomized, Double-Blind, Placebo-Controlled Phase 2 Trial of TransCon PTH in Adult Hypoparathyroidism. The Journal of Clinical Endocrinology & Metabolism.

[cit26] Khan Aliya A, Rubin Mishaela R, Schwarz Peter, Vokes Tamara, Shoback Dolores M, Gagnon Claudia, Palermo Andrea, Marcocci Claudio, Clarke Bart L, Abbott Lisa G, Hofbauer Lorenz C, Kohlmeier Lynn, Pihl Susanne, An Xuebei, Eng Walter Frank, Smith Alden R, Ukena Jenny, Sibley Christopher T, Shu Aimee D, Rejnmark Lars (2022). Efficacy and Safety of Parathyroid Hormone Replacement With TransCon PTH in Hypoparathyroidism: 26-Week Results From the Phase 3 PaTHway Trial. Journal of Bone and Mineral Research.

[cit27] Clarke Bart L, Khan Aliya A, Rubin Mishaela R, Schwarz Peter, Vokes Tamara, Shoback Dolores M, Gagnon Claudia, Palermo Andrea, Abbott Lisa G, Hofbauer Lorenz C, Kohlmeier Lynn, Cetani Filomena, Pihl Susanne, An Xuebei, Smith Alden R, Lai Bryant, Ukena Jenny, Sibley Christopher T, Shu Aimee D, Rejnmark Lars (2024). Efficacy and Safety of TransCon PTH in Adults With Hypoparathyroidism: 52-Week Results From the Phase 3 PaTHway Trial. The Journal of Clinical Endocrinology & Metabolism.

